# Centralized bundle generation in auction-based collaborative transportation

**DOI:** 10.1007/s00291-018-0516-4

**Published:** 2018-03-29

**Authors:** Margaretha Gansterer, Richard F. Hartl

**Affiliations:** Department for Business Administration, University of Vienna, Oskar-Morgenstern-Platz 1, 1090 Vienna, Austria

**Keywords:** Logistics, Collaborations, Transportation, Combinatorial auctions, Genetic algorithms

## Abstract

In horizontal collaborations, carriers form coalitions in order to perform parts of their logistics operations jointly. By exchanging transportation requests among each other, they can operate more efficiently and in a more sustainable way. This exchange of requests can be organized through combinatorial auctions, where collaborators submit requests for exchange to a common pool. The requests in the pool are grouped into bundles, and these are offered to participating carriers. From a practical point of view, offering all possible bundles is not manageable, since the number of bundles grows exponentially with the number of traded requests. We show how the complete set of bundles can be efficiently reduced to a subset of attractive ones. For this we define the Bundle Generation Problem (BuGP). The aim is to provide a reduced set of offered bundles that maximizes the total coalition profit, while a feasible assignment of bundles to carriers is guaranteed. The objective function, however, could only be evaluated whether carriers reveal sensitive information, which would be unrealistic. Thus, we develop a proxy for the objective function for assessing the attractiveness of bundles under incomplete information. This is used in a genetic algorithms-based framework that aims at producing attractive and feasible bundles, such that all requirements of the BuGP are met. We achieve very good solution quality, while reducing the computational time for the auction procedure significantly. This is an important step towards running combinatorial auctions of real-world size, which were previously intractable due to their computational complexity. The strengths but also the limitations of the proposed approach are discussed.

## Introduction

**Fig. 1:**
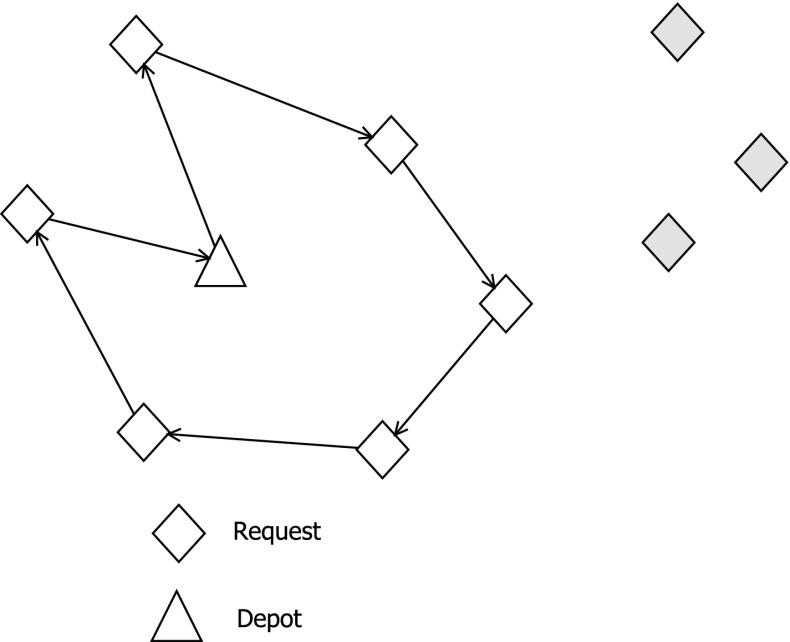
Tour of a carrier with 6 requests. Including one of the new (gray) requests will extend his tour length considerably, but will probably not be profitable. However, if he or she additionally wins the second or even third request, the extra travel cost will probably be compensated by the extra profit

In the highly competitive transportation industry, carriers need to aim for a maximum level of efficiency in order to stay in business. Fierce competition brings prices down; therefore, profit margins of carriers have declined to an extremely low level. By increasing efficiency, collaborations also serve ecological goals. Thus, public authorities are encouraging companies to collaborate. They aim at reduced road congestion, noise pollution, and emissions of $\mathrm{CO}_2$ and other harmful substances. The city of Zurich, for instance, is funding a research project aiming at improved cooperation between different transport companies by an IT-based collaboration platform (Schmelzer 2014). To approach the goal of maximized efficiency, carriers can, for instance, participate in collaborative networks and trade their transportation requests among each other. This is commonly done by using auction-based exchange systems. In *combinatorial auctions*, requests are not traded individually but are combined into packages, i.e., bundles. The main reason for this is that a specific bundle of requests might have a different value to the partners than the sum of the individual requests. An example for this is displayed in Fig. 1.

A bidding carrier receives the full bundle if the bidding price is accepted. If the bid is rejected, none of the items contained in the package is transferred. This eliminates the risk of only obtaining a subset of requests which does not fit into the current request portfolio, because, for instance, the requests are geographically distant from the other requests of the carrier.

A combinatorial transportation auction typically follows a 5-phase procedure (Berger and Bierwirth 2010):

1. Carriers decide which requests to put into the auction pool
2. Auctioneer generates bundles of requests and offers them to the carriers
3. Carriers give their bids for the offered bundles
4. Winner Determination Problem (WDP): Auctioneer allocates bundles to carriers based on their bids
5. Gained profits are distributed among the carriers.

The success of such a collaboration clearly depends on the attractiveness of bundles that are offered to the carriers. Thus, a good selection of traded requests (Phase 1) is crucial in order to yield profitable auctions (cf. Gansterer and Hartl 2016). However, based on the requests that have been submitted to the auction pool, the auctioneer has to decide on the bundling of requests such that attractive packages can be offered to the carriers (Phase 2). From a practical point of view, offering all possible bundles is not manageable, since the number of bundles is $2^n -1$, where *n* is the number of requests that are traded. Alternatively, the bundling problem can be moved to the carriers themselves. In such a setting, the auctioneer would offer the set of requests without grouping them to bundles. The carriers then give their bids on self-created packages of requests (Ackermann et al. 2011). The obvious drawback of this approach is that the auctioneer receives bundles with a high attractiveness for each individual carrier, but faces low flexibility in the WDP. In particular, if carriers submit overlapping bundles. Additionally, if requests that are not included in the carriers’ bundles have to be returned to their owners, an outsourcing option has to be provided. Otherwise, carriers who acquire new requests but cannot auction off some of their existing ones will violate their capacity constraints. Berger and Bierwirth (2010) let the auctioneer build the bundles. They build the full bundle set (i.e., the power set of the requests), but in order to reduce the complexity, they assume that carriers are not allowed to submit more than one request to the pool. In this sense, the bundling problem is at least one of the less understood parts of the auction phases listed above. The aim of the current paper is to fill this gap.

We show that the bundling can be made by the auctioneer, while the number of bundles can be limited to a reasonably sized subset of possibly attractive ones. Since auctioneers typically do not have carrier-specific information, attractiveness has to be evaluated under incomplete information. Although carriers do not have to reveal sensitive information, our approach guarantees to find a feasible assignment of bundles to carriers.

We define the theoretical model, which we refer to as the Bundle Generation Problem (BuGP).

Its aim is to provide the set of offered bundles that maximizes the total marginal profit. Since the objective function could only be evaluated whether carriers reveal sensitive information, we develop a proxy for the objective function. The proxy is used to evaluate the attractiveness of bundles under incomplete information. This evaluation is then used in a GA-based framework that aims at producing attractive and feasible bundles, such that all requirements of the BuGP are met.

In the following, we want to highlight the contribution of our study:

- We introduce BuGP, in which the objective is to find the set of offered bundles that maximizes the total marginal profit.
- Using a proxy for the objective function in an GA-based framework, we are able to produce attractive and feasible sets of offered bundles.
- By the limitation of the bundle pool, the computational effort is reduced such that even real-world sized combinatorial auctions can be run and analyzed. To the best of our knowledge, we are the first to show that the enormous complexity of the bundling phase of combinatorial transportation auctions can be efficiently reduced to a reasonable level. The approach, however, comes with some limitations like the low freedom of action for the participants.
- We provide interesting findings regarding collaboration profits and runtimes, if the auction procedure works with limited instead of complete sets of offered bundles. In particular, we show that with only a little loss in solution quality, instances can be solved in a fraction of the computational time.

The remainder of the paper is organized as follows. Section 2 provides a literature review. Details on the combinatorial auction are given in Sect. 3. We design the investigated decision problem in Sect. 4. Details on the solution approach and the computational study are presented in Sects. 5 and 6, respectively. Conclusions and further research are summed up in Sect. 7.

## Literature review

The transportation industry has expanded rapidly in a highly competitive environment. Logistics companies with insufficient transport capacities are forced to make a selection of customers that they serve with their own fleet. Remaining customer requests can, for instance, be offered to collaboration partners in order to increase total profits.

Collaborations in freight logistics have been extensively studied in the past years (e.g., Ackermann et al. 2011; Dahl and Derigs 2011; Puettmann and Stadtler 2010; Krajewska et al. 2008; Ergun et al. 2007a, b; Cruijssen et al. 2007; Krajewska and Kopfer 2006). Thus, in our literature review we focus on the application of combinatorial auctions in the field of vehicle routing.

Combinatorial auctions have been shown to be effective mechanisms to allocate transportation requests by, e.g., Ledyard et al. (2002), Sheffi (2004), and Berger and Bierwirth (2010). General information on combinatorial auctions and combinatorial auction design can be found in de Vries and Vohra (2003) and Pekeč and Rothkopf (2003), respectively. These mechanisms are of particular relevance in the pickup and delivery market, where shipments from several different customers can be moved on a single vehicle (Li et al. 2016; Gansterer et al. 2016).

A decentralized request exchange mechanism for less than truckload (LTL) transportation is proposed by Wang and Kopfer (2014), where carriers can iteratively generate and submit new routes based on the feedback information from an agent.

Collaboration among independent freight forwarding entities, who can either self-fulfill or subcontract orders to external freight carriers, is investigated by Krajewska and Kopfer (2006). It is assumed that the freight forwarders participate in coalitions in order to gain additional profit. A model which is based on combinatorial auction theory as well as on the operations research game theory is presented.

A combinatorial exchange mechanism exploiting synergies for transportation routes of various carriers is presented by Schwind et al. (2009). Ackermann et al. (2011) discuss various goals for a combinatorial request exchange in freight logistics aiming at improving usefulness in a practical environment of LTL carriers.

An intuitive approach to reduce the auction’s complexity is to limit the number of requests in the pool. Li et al. (2015) do not allow the carriers to trade more than one request. Xu et al. (2016) show auction mechanisms for the truckload carrier collaboration problem with bilateral lane exchange. Again carriers offer only one lane. Also in the multi-agent framework presented by Dai and Chen (2011), there is only one request traded per auction round. Carriers act as auctioneers when they want to outsource a request to other carriers, whereas they act as bidders when they want to acquire a request from other carriers.

Some authors assume that bundles are built not by the auctioneer, but by the carriers themselves (Wang and Xia 2005; Buer 2014; Triki et al. 2014). They refer to this as the *Bid Generation Problem*. These studies provide decision support for carriers in the sense that good selection methods are developed. They do, however, not take the total collaboration profit into account. Also a feasible solution for the WDP cannot be guaranteed.

The complexity of the bidding phase can also be reduced by performing multi-round (or iterative) auctions (de Vries and Vohra 2003). These are generally intended to offer subsets of the traded items in multiple rounds. Previously gained information can be used to compose the setting for the next round. By this, the bidders are never faced with the full complexity of the auction pool. The major drawback is that the auctioneer has to be equipped with sophisticated rules on the selection of offered items in each round as well as an efficient learning procedure. A multi-round price-setting-based combinatorial auction approach for carrier collaboration is proposed by Dai et al. (2014). In such a *price-setting* mechanism, the auctioneer, however, does not offer bundles but he or she sets prices. Carriers then select requests that they are willing to serve for the given price.

Chen et al. (2009) propose combinatorial auctions for truckload procurement that enable the complete set of all possible bids to be considered implicitly, without placing the corresponding burden of an exponential number of bids on either the bidders or the auctioneer. Their study focuses on transportation procurement, where they argue that privacy issues between shippers and carriers might be overcome. Our method is intended for horizontally collaborating carriers, for whom information exchange with competitors is of course undesirable.

To the best of our knowledge, we are the first to introduce an approach to reduce the enormous complexity of the bundle pool in combinatorial transportation auctions, without the need for carriers to reveal private information. In contrast to traditional approaches, our mechanism demands a relatively powerful auctioneer, who is in charge of building bundles and gives only low freedom of action to the participants. From the carriers perspective, this might be seen critical. However, the computation results emphasize the strength of our approach. Additionally, we discuss game-theoretical aspects in Sect. 3.

## The combinatorial auction

We assume carriers to serve LTL paired pickup and delivery requests, which means that each request is associated with a given origin and destination. A carrier starts at the depot, visits a given set of pickup and delivery nodes and returns to the depot again. The objective is to minimize total travel time. This problem belongs to the class of Vehicle Routing Problems with Precedence Constraints. Parragh et al. (2008) refer to this problem as the single vehicle case of the Vehicle Routing Problem with Pickups and Deliveries (SPDP). Berbeglia et al. (2007) classify it as one-to-one pickup and delivery problem. The mathematical model of the underlying routing problem for a single carrier is given in Gansterer and Hartl (2016).

Each carrier is supposed to select requests for the auction pool. This selection is done based on distances between requests. This means that a carrier submits a given number of requests to the pool that have a very low travel distance among them. It has been shown by Gansterer and Hartl (2016) that this selection rule typically leads to very good results.

Requests in the auction pool are packaged to bundles by the auctioneer. Details on the bundling procedure are given below (see Sect. 4). Bundles are then offered to the carriers.

Bids are generated based on the carriers’ marginal profit, which is the difference of the profit including and excluding the bundle in the tour. Hence, for each bid a routing problem has to be solved. We do this heuristically using the double insertion heuristic (Renaud et al. 2000) followed by an improvement phase based on the well-known 3-opt algorithm (Lin 1965). Since the carriers have tour length restrictions, we have to include this information into the bidding process. A carrier does not submit a bid for a bundle that cannot be inserted into his tour. However, as long as the tour length restrictions are not violated a carrier has to submit a bid for each offered bundle (Berger and Bierwirth 2010; Gansterer and Hartl 2016).

Based on the bids, the auctioneer has to find an allocation of bundles to carriers such that the total network profit is maximized. This is the well-known WDP (see, e.g., Remli and Rekik 2013; de Vries and Vohra 2003), where we assume that each carrier wins at most one bundle. This guarantees that none of the carriers gets requests that he or she cannot handle. The mathematical model is formulated as follows (based on Gansterer and Hartl 2016):

*C*: Set of bidders/carriers, $c\in C$
*R*: Set of requests, $r\in R$
*B*: Set of offered bundles, $b\in B$
$$p_\mathrm{bc}$$
Price carrier *c* is willing to pay for bundle *b*
*P*: Matrix of all prices
$$\pi (B,P)$$
Total marginal profit after solving the WDP with *B* and *P*
$$W_\mathrm{br}$$
Parameter indicating whether request *r* is included in bundle *b* or not
$$Q_\mathrm{bc}$$
0/1 parameter indicating whether carrier *c* submitted a bid for bundle *b* or not
$$v_\mathrm{bc}$$
Decision variable indicating whether bundle *b* is allocated to carrier *c* or not

1 $$\begin{aligned}&\pi (B,P)=\max \sum _c \sum _b p_\mathrm{bc}v_\mathrm{bc} \end{aligned}$$

2 $$\begin{aligned}&\sum _b v_\mathrm{bc}\le 1\quad \quad \forall c\in C\end{aligned}$$

3 $$\begin{aligned}&\sum _c v_\mathrm{bc}\le 1\quad \quad \forall b\in B\end{aligned}$$

4 $$\begin{aligned}&v_\mathrm{bc}\le Q_\mathrm{bc}\quad \quad \forall b\in B, c\in C\end{aligned}$$

5 $$\begin{aligned}&\sum _b\sum _c v_\mathrm{bc}W_\mathrm{br}=1\quad \quad \forall r\in R\end{aligned}$$

6 $$\begin{aligned}&{v_\mathrm{bc}}\in \left\{ 0,1\right\} \quad \quad \forall b\in B, c\in C \end{aligned}$$

Objective function (1) maximizes the total marginal profit. Each carrier can only win one or no bundle (2). Constraint (3) ensures that each bundle can only be allocated once. A carrier can only win a bundle if he or she submitted a bid price for it (4). We have to ensure that each request is allocated exactly once. This is done by Constraint (5). Obviously, we formulate the WDP as a *set partitioning problem*, while in a standard combinatorial auction the WDP is assumed to be a *set packing problem* (Pekeč and Rothkopf 2003). Depending on the area of application, it is recommendable to use a *set covering problem* formulation (Buer and Pankratz 2010), which reduces the risk of facing infeasible problems. However, this is not applicable in our study, since we assume that each request can only be executed by one carrier. We avoid infeasibility by letting the auctioneer build bundles that are feasible solutions of the set partitioning problem (see Sect. 4). Finally, Constraint (6) defines that the decision variables are binary.

The allocation of bundles is executed if a solution with an increased total network profit is found. The lower bound for the total network profit is the initial situation. It is guaranteed that in the end each carrier has at least his initial profit, since the total payments can be redistributed such that individual losses are compensated. For more detailed information on the auction-based framework, we refer to Berger and Bierwirth (2010). It has also been applied in Gansterer and Hartl (2016).

Using an auction mechanism, we should aim to realize the following four standard properties (Xu et al. 2016): (i) efficiency, (ii) budget balance, (iii) individual rationality, and (iv) incentive compatibility. The proposed mechanism is efficient, in that the WDP maximizes value creation for the traded bundles. Budget balance implies that the auctioneer cannot make a loss, which we can guarantee. Our mechanism is individual rational, since each participant has a nonnegative profit. However, since the WDP uses each participants bid prices, we cannot guarantee that strategic behavior, i.e., untruthful bidding, does not increase a bidders profit on the cost of others. Thus, the proposed mechanism is not incentive compatible, which would imply that there are no any incentives for participants to bid untruthfully. Second price auctions (Vickrey 1961) or more general Vickrey–Clarke–Groves auctions (Vickrey 1961; Clarke 1971; Groves 1973) are known to be incentive compatible. However, they are impractical and rarely used in practice (Pekeč and Rothkopf 2003). While they are incentive compatible for the bidding process, they might still be subject to several kinds of cheating, which in our case might be the selection of request that are offered for trading. Pekeč and Rothkopf (2003) claim that they are unsustainable in realistic dynamic environments in which the revelation of the bidders values has consequences beyond the auction.

Moreover, double or two-sided auctions, where both buyers and sellers submit bids, are among the most prevalent forms of economic transactions (Kojima and Yamashita 2017). In our case, complexity is increased by the fact that all participants can be sellers and buyers at the same time, and that transportation requests have synergies among them. To the best of our knowledge, there is no mechanism available in the literature that realizes all desirable properties for such markets.

However, while we cannot guarantee incentive compatibility, a profitable untruthful bidding strategy is not easy to find. Due to the synergies among transportation requests, and the fact that a carrier does not have to sell his or her requests, but can also serve them by his or own, the outcome of strategic bidding is hard to predict. We emphasize this by a preliminary computational study, where we observe that a natural approach of untruthful bidding is not always the dominant strategy. For this, we use test instances O1, O2, O3, which are described in Sect. 6. There are 3 carriers (C0, C1, C2), where only C0 cheats by increasing his or her bid by factor $\alpha$, which we assume to be 1.1, 1.4, or 1.7. Obviously, this increases the probability to win a bundle. Furthermore, this strategy increases the share of the gained total payments. This is because the carrier will also increase bids on his or her own requests, and these bids are used by the auctioneer to determine how much this carrier has to gain such that she reaches at least his or her initial profit. Since these increased bids come with disadvantage to be forced to pay higher prices, we let the cheating carrier only bid untruthful for requests that are not in the close proximity of his or her own depot. This is based on the assumption that it does not pay off to bid on close requests, that will be won anyway. This closeness is denominated as $\beta$, which is the relative distance to the depot. The cheating carrier bids untruthful if the center between pickup and delivery point is more than $\beta$ away from the depot. We assume $\beta$ to be 0.5 or 0.7. The results are summarized in Table 1.

**Table 1:** Average percentage loss (negative numbers) or additional profit (positive numbers) of carriers *C*0 (cheater), *C*1 and *C*2 compared to truthful bidding

$\alpha$	$\beta$	*C*0 (%)	*C*1 (%)	*C*2 (%)
1.1	0.5	$-$ 1.44	$-$ 2.52	0.18
1.1	0.7	$-$ 12.01	$-$ 5.26	2.43
1.4	0.5	$-$ 3.57	1.30	$-$ 5.35
1.4	0.7	$-$ 8.63	1.50	1.03
1.7	0.5	$-$ 7.14	0.58	$-$16.55
1.7	0.7	$-$ 6.92	$-$ 9.95	$-$ 4.23

We see that the cheating carrier (C0) on average is worse-off compared to truthful bidding. In some instances untruthful bidding of carrier C0 even increases the final profit of the competitors. While no general conclusions can be drawn from this, it shows that a profitable cheating strategy is not straight forward and potentially does not even exist.

The proposed mechanism uses a very simple profit sharing method, where individual losses are compensated and the additional surplus is shared equally. The commonly recommended profit sharing method is the Shapley value (Guajardo and Rönnqvist 2016). For calculating Shapley values, carriers would have to reveal sensitive information like their complete set of customers. Given the realistic assumption, that carriers are not willing to reveal this kind of information, Shapley value is not applicable in this study. Several adapted versions, where only the traded requests are considered might be a attractive alternatives to the proposed profit sharing method. However, this is not in the scope of this study, since we focus on how to reduce complexity in order to make previously intractable auctions tractable. Readers interested in profit sharing or cost allocations methods are referred to Guajardo and Rönnqvist (2016). Please note that this section describes the whole process of the combinatorial auction (Phases 1–5 given in Sect. 1). In the following section, we give detailed information on the bundling process (Phase 2).

## The bundle generation problem

In order to yield high collaboration profits, the auctioneer has to provide attractive bundles of requests to the carriers. There is little literature on the bundling phase of combinatorial transportation auctions (see Sect. 1). In previous research, it is, however, assumed that all possible bundles are offered (e.g., Berger and Bierwirth 2010; Gansterer and Hartl 2016). Regarding practical applications, this can hardly be handled. The number of bundles is $2^n -1$, where *n* is the number of requests that are traded. Since it is assumed that carriers have to calculate their bids for all bundles that are offered by solving an NP-hard optimization problem, forcing them to evaluate $2^n -1$ bundles is obviously problematic. Thus, the auctioneer has to limit the number of bundles to a reasonable number of possibly *attractive* ones. Since it is assumed that carriers are not willing to reveal sensitive data, the auctioneer has only incomplete information on the future bid prices for offered bundles. The BuGP is formalized in the following mathematical model:

$${\mathbf {E}}_\sigma $$
expectation operator with respect to scenario $\sigma$
*P*: matrix of prices in scenario $\sigma$
$${\mathcal {P}}(R)$$
power set of *R*
*L*: maximum number of offered bundles

7 $$\begin{aligned}&\max \quad {\mathbf {E}}_{\sigma }\ \pi (B,P_{\sigma }), \end{aligned}$$

8 $$\begin{aligned}&\vert B\vert \le L, \end{aligned}$$

9 $$\begin{aligned}&B \subset {\mathcal {P}}(R). \end{aligned}$$

Objective function (7) maximizes the objective value of the WDP solved for bundle set *B*. In this model, we assume that the auctioneer does not have information on the prices, but can at least identify a set of possible pricing scenarios $\sigma$. Constraint (8) restricts the number of offered bundles to a given limit *L*. *B* is a subset of the power set of *R*.

We assume that the carriers are not willing to reveal sensitive information like their existing customers, routes or available capacities. Thus, pricing matrices can hardly be determined. The auctioneer, however, has to offer bundles that can be combined to a feasible assignment in the WDP. This implies that there are no unassigned requests, while carrier capacities are not exceeded. Thus, the auctioneer has to deal with feasibility *and* attractiveness of bundles under incomplete information.

To increase the expected objective value of the WDP, we propose an algorithm that does not focus on single bundles, but constructs *candidate solutions*. Candidate solutions are combinations of non-overlapping bundles including all requests. This is in line with a renowned concept in the field of *matheuristics*, which is classified by Archetti and Speranza (2014) as *Branch-and-price/column generation-based matheuristics*. It has been successfully applied to routing problems by, e.g., Cacchiani et al. (2014), Parragh and Schmid (2013), Villegas et al. (2013), Schmid et al. (2009). The idea is to generate sets of feasible routes (e.g., heuristically generated VRP solutions), which are then passed to a restricted master problem (e.g., a set partitioning problem), that is solved to optimality (Dörner and Schmid 2010). It has been shown that the concept of passing complete VRP solutions to restricted master problems offers flexibility to find very good solutions for underlying complex routing problems.

In our problem, a candidate solution is a set of bundles that covers all requests. We intentionally do not refer to these sets as *feasible bundles*, since we want to emphasize that the auctioneer does not know carriers’ capacities. Thus, the auctioneer builds bundles not knowing whether they are feasible in terms of capacity restrictions. The number of bundles in a candidate solution is limited by the number of carriers, since we assume that each carrier gets at most one bundle (see Sect. 3).

Bundles included in candidate solutions are offered to the carriers in the bidding phase. The WDP, which is defined as a set partitioning problem (see Sect. 3), then finds an optimal assignment of bundles to carriers. However, in our problem it is not possible to generate sets of feasible bundles, i.e., candidate solutions, based on real routing costs or carriers’ capacities. These are not known to the auctioneer. In order to overcome this problem, we propose a heuristic that approximates the attractiveness of bundles (i.e., the marginal profit) based on geographical information. It is used as fitness function in a GA that produces candidate solutions.

**Fig. 2:**
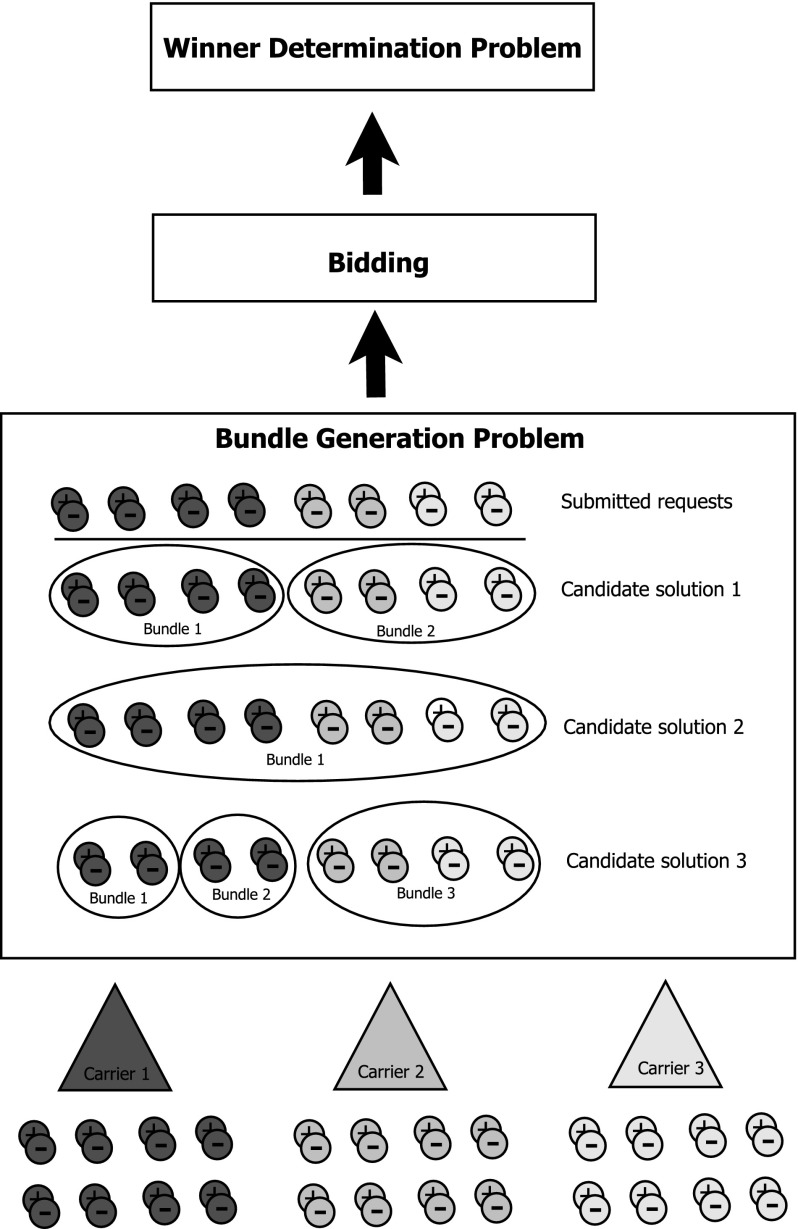
Illustration of the BuGP for 3 carriers, who are submitting requests to the auction. Requests that are not submitted (below triangle) are not revealed to the auctioneer

In Fig. 2, we illustrate the BuGP for 3 carriers. Each carrier puts some request into the pool, which are then used by the auctioneer to build candidate solutions. Requests kept by the carriers, i.e., those that are not submitted to the pool, are not known to the auctioneer. Thus, candidate solutions have to be built under incomplete information. All bundles are offered to the carriers in the bidding phase. Finally, the WDP finds an optimal assignment of bundles to carriers.

In the following section, we present a GA-based framework that is used by the auctioneer to generate attractive bundles.

## Solution approach

It is assumed that carriers are not willing to share private information. Thus, the matrix of prices *P* used in (7) cannot be determined by the auctioneer. We therefore develop a proxy function that approximates the attractiveness of bundles under incomplete information. We only consider obvious characteristics of the submitted requests and try to combine them to bundles that seem to be attractive from a general point of view. Our GA-based framework produces sets of offered bundles that meet all requirements of the BuGP.

### Proxy for the objective function

The proxy is based on the intuitive assumption that good bundles are (i) dense, (ii) isolated from other bundles, and (iii) include requests that can be visited within short travel time.

**Fig. 3:**
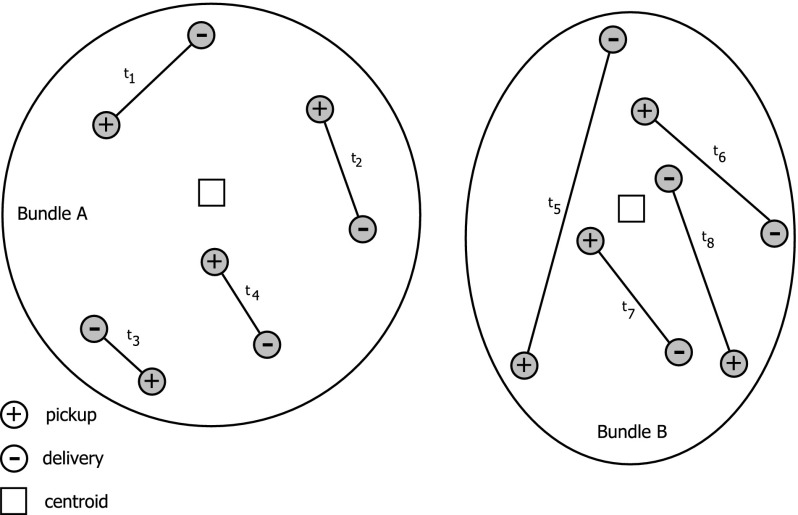
Example of a bundle with relatively low density (bundle A) and relatively high density (bundle B)

We define the following parameters for each bundle:

- *Centroid* this is the centroid of the request’s centers, where the center of request is the midpoint between pickup and delivery location. For this, the centers are weighted with the length of their request, which is the direct travel distance $t_r$ between pickup and delivery of request *r*.
- *Radius* this is the average distance of all points in the bundle (pickup and delivery) to the bundle’s centroid. $rad_E$ is the radius of bundle *E*.
- *Density* we calculate the density by the average direct travel distance $t_r$ of all requests *r*, divided by the maximum of the distances of all requests to the bundle’s centroid. The distance of a request to the bundle’s centroid is determined by the sum of the distances of its pickup point and its delivery point to the centroid. In Fig. 3, we display both an example for a bundle with relatively low and relatively high density. We see that in bundle A travel distances $t_r$ of the requests are relatively short, while the maximum distance from all requests to the centroid is relatively long. On the other hand, in bundle B the travel distances $t_r$ for the requests are rather long, while the maximum distance from the requests to the centroid is relatively short.
- *Isolation* This parameter approximates the separation from other bundles. For calculating a separation value $sep_\mathrm{EF}$ between two bundles *E* and *F*, we first calculate the distance between their centroids ($dist_\mathrm{EF}$), as well as their radii $rad_E$ and $rad_F$. The separation value $sep_\mathrm{EF}$ is then determined as follows:
  10 $$\begin{aligned} sep_\mathrm{EF} = \frac{dist_\mathrm{EF}}{\max (rad_E,rad_F).} \end{aligned}$$
  We define the isolation of a bundle as the minimum of all its separation values.
- *Tour length* This is the total travel distance needed to visit all requests in a bundle. Since finding the optimal solutions for all bundles is too time consuming, the tour length is approximated using an algorithm proposed by Renaud et al. (2000). Initial solutions are built by a double insertion heuristic. We improve them using the well-known 3-opt algorithm (Lin 1965).

In an extensive study, we investigated many different proxies. For instance, we tried to include additional geographic information like the locations of carriers’ depots. Also different weighting factors for the proxy’s components have been evaluated. However, it turned out that evaluating a candidate solution *s* based on the following formula yields the best results:

11 $$\begin{aligned} \frac{{\displaystyle \min _{b\in B_s}}(i_\mathrm{b})*{\displaystyle \min _{b\in B_s}}(d_\mathrm{b})}{{\displaystyle \max _{b\in B_s}}(t_\mathrm{b})*m} \end{aligned}$$

where $i_\mathrm{b}$, $d_\mathrm{b}$, and $t_\mathrm{b}$ are isolation, density, and tour length of bundle $b\in B_\mathrm{s}$. *m* is the number of bundles in the solution. Thus, for evaluating a candidate solution, its bundles with the minimum isolation value, minimum density value, and maximum tour length are decisive. By including *m*, we take into account that candidate solutions including many (and therefore smaller) bundles will naturally have shorter tour lengths than candidate solutions with less bundles.

To give an impression on the impact of the proxy function, we present results of two alternative proxies. The first one aims for a low isolation rather than a high isolation. We refer to this as *Alternative* 1 (*A*1). The second one (*A*2) maximizes isolation and tour length, while density is minimized. Intuitively, both of the seem promising, since they might provide bundles that can easily be integrated into existing tours. For this comparison, we use test instances O1, O2, O3, which are described in Sect. 6. Results are summed up in Table 2.

**Table 2:** Average deviation in solution quality (based on the total profit) if proxy function *A*1 or *A*2 is used instead of the proposed one

# Bundles	*A*1 (%)	*A*2 (%)
100	$-$ 25.3	$-$ 23.1
500	$-$ 29.1	$-$ 18.9

*# Bundles* give the number of traded bundles

We observe that the used proxy function has a significant impact on solution quality. Using *A*1 decreases the total profit by up to 29.1%. Also *A*2 is clearly dominated by the proposed proxy.

### GA-based framework

To produce the final set of offered bundles, we use a GA-based framework. GA is a metaheuristic working with a set of solutions (called population), which is manipulated, by evolution-inspired methods like crossover and mutation. By these operators, it produces new populations with higher evaluated solutions. A problem-specific fitness function is used to assess the value of a solution. A decoding scheme is used to represent solutions as a string of numbers (Goldberg 1989). GA has been successfully used for many decades and is still one of the most successful metaheuristics in vehicle routing (e.g., Cattaruzza et al. 2014; Vidal et al. 2014).

The method produces a population of candidate solutions, where each candidate solution consists of a set of bundles, which are subject to the properties described in Sect. 4. The bundles from the final population are then offered to the participating carriers.

**Fig. 4:**
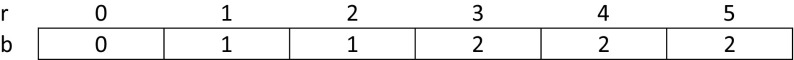
GA decoding for a candidate solution *s* with 6 requests and 3 bundles. Request 0 is in bundle 0, requests 1 and 2 are in bundle 1, and requests 3–5 are in bundle 2

*Decoding* An individual is a string of $\vert R\vert$ numbers, where each entry $b_r$ gives the bundle a request *r* is assigned to. Figure 4 displays an example for a candidate solution with 6 requests and 3 bundles.

We use a normalized representation of candidate solutions, in order to identify duplicates within a population. For this, it is assumed that the bundle that includes the first request gets number 0. The bundle that gets the request that (i) has the lowest number and (ii) is not included in bundle 0, gets number 1, and so on.

**Fig. 5:**
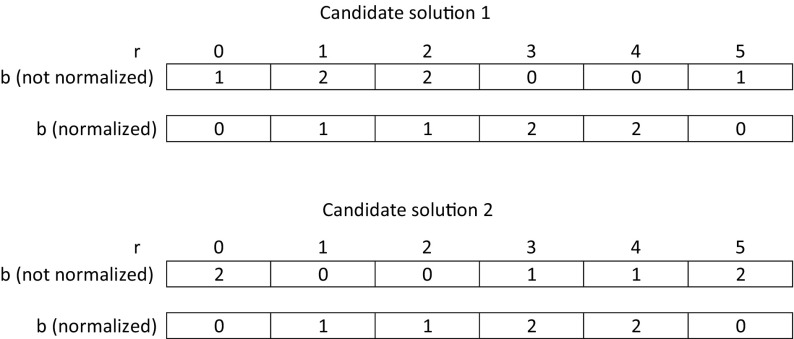
Two candidate solutions before and after normalization

All following bundle numbers are assigned in increasing order. By this we can guarantee that any two solutions can be directly compared, and redundant individuals can be eliminated from the population. We refer to this as the *normalized*, i.e., renumbered, representation of a candidate solution. For better understanding, Fig. 5 shows two identical candidate solutions before and after normalization. We see that only after normalization, the individuals can be easily identified to be identical.

The initial population consists of a randomly generated set of candidate solutions. Each of them consists of 1 to *n* bundles. When building a new generation of individuals, the best individuals of the previous generation are passed over without any change. This is what is called *elitism*. Individuals for crossover are chosen by a roulette wheel based on their fitness rank. There are two types of crossover. With 50% probability, the one or the other type is chosen. With a given probability, newly generated individuals are mutated. The new population is then filled up with mutated individuals of the previous generation. Population diversity is considered by eliminating redundant individuals.

After extensive tests with various crossover variants, the following two turned out to be the most effective ones:

*Crossover 1* For each request, the corresponding bundle is randomly chosen from parent A or B. This corresponds to the uniform crossover of Michalewicz (1996), where only one child is produced.

**Fig. 6:**
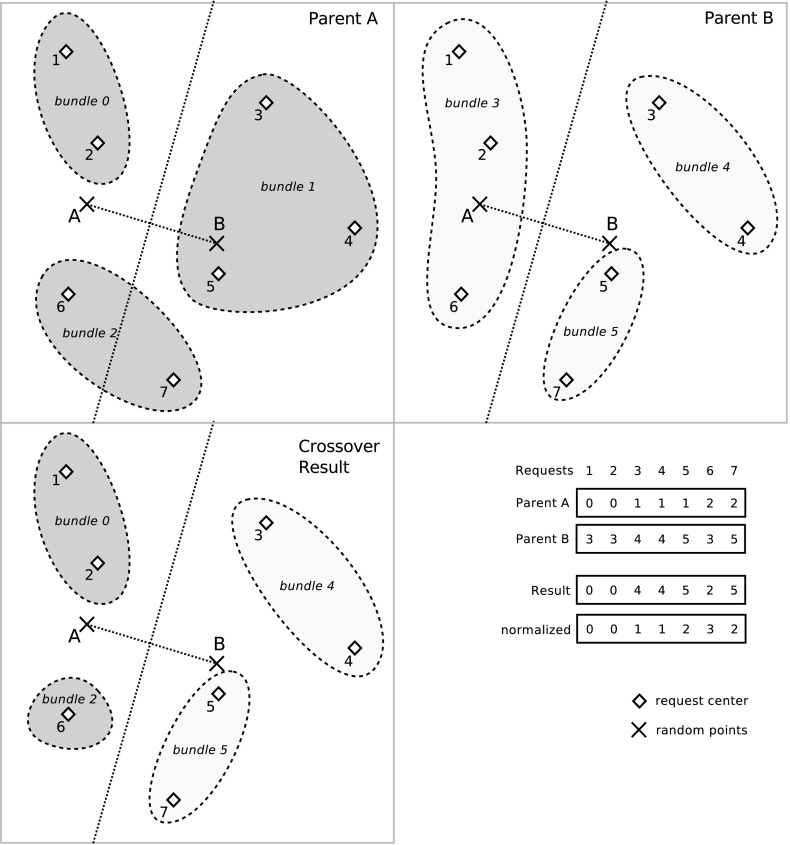
Example for crossover 2 with 7 requests. Parents A and B consist of 3 bundles each. In the crossover result, 4 bundles are formed. For the sake of comprehensibility, the string of parent B is not displayed in the normalized form but with an offset of 3

*Crossover 2* In this operator, we try to keep potentially good parts of existing bundles by combining the parents using geographic information. First, we calculate the center of each request, which is the midpoint between pickup and delivery location. Then, we randomly generate two points (A and B) in the plane. If the center of a request is closer to A, it is assigned to the bundle given in parent A, but if it is closer to B, it gets the bundle given in parent B. An example of crossover 2 is illustrated in Fig. 6 for 6 requests. If the new solution consists of too many bundles, two randomly chosen bundles are merged.

*Mutation* We use four mutation operators. After crossover one of the four operators is applied with a given probability:

1. *Move* a random number of randomly chosen positions is changed. However, the number of available bundles is not increased.
2. *Create* a new bundle is created. We randomly chose one request and assign it to the new bundle. If by this the maximum number of bundles is exceeded, i.e., if there are more bundles than carriers (see Sect. 4), two randomly chosen bundles are merged.
3. *Join* two randomly chosen bundles are merged.
4. *Shift* for each of the given bundles in the candidate solution, the centroid is calculated. Then, requests are assigned to bundles according to their closeness to the bundle’s centroids.

*Fitness function* The fitness of the candidate solutions is determined by the proxy presented in Sect. 5.1.

*Bundle pool generation* The GA stops if a given number of generations has been built. In the auction process, there is a limit *L* on the number of bundles that are offered to the carriers. For composing the set of offered bundles, the candidate solutions in the final GA population are sorted according to their fitness. Starting at the best solution, for each solution all its bundles are added to the auction pool until the limit *L* is reached. Although candidate solutions are unique, it might of course happen that the same bundle appears more than once in different candidate solutions. If a bundle has already been submitted to the auction pool, it is not submitted again.

Each offered bundle has to be part of at least one candidate solution (see Sect. 4). Since a candidate solution has to be considered with all its bundles or not at all, we have to allow the limit of bundles to be slightly exceeded.

Since carriers’ capacities are assumed to be hidden, our algorithm can of course not guarantee that enough feasible bundles (i.e., bundles that meet with the capacity restriction of at least one carrier) are generated. If the auctioneer does not offer any combination of feasible bundles (i.e., a realizable candidate solution), the WDP becomes infeasible. However, to avoid this, the auctioneer can simply add bundles that include exactly the requests a carrier has offered for trading. By this, feasibility of the WDP is guaranteed.

*Preceding experiments* In the course of our research we tested various approaches. The most relevant of these were clustering by the k-means algorithm (Lloyd 1982) using different seeds and building clusters based on the minimal tour lengths. The k-means approach produces geographically dense bundles but does not take tour lengths into account. The tour length approach on the other hand has the tendency to produce overlapping bundles. This observation was the basis for generating the GA approach with the specific fitness function presented here. The results of these approaches were by far worse than those found by the GA-based framework. We therefore do not report them here.

## Computational study

In our computational study, we consider different scenarios in terms of (i) degree of customer overlaps and (ii) distance of requests to the carriers’ depots. Following Berger and Bierwirth (2010), we use self-created random instances. We assume that 3 carriers operate in overlapping but not identical customer regions. There are 3 types of instances depending on the degree of customer area overlaps (O1_xx, O2_xx, O3_xx). For each instance, we generate equidistant carrier depots with a distance of 200. Requests are randomly generated within a radius of 150 (O1), 200 (O2), and 300 (O3). Each carrier initially holds either 10 or 15 requests. We refer to these scenarios as O1_10, O1_15, O2_10, O2_15, O3_10, and O3_15, respectively. We generate 20 instances for each scenario. The instances are publicly available (http://prolog.univie.ac.at/research/BundlingInst/Bundling_instances.zip). All experiments are coded in C++ and executed single threaded on an Intel Core i5-3570 3.4GHz computer.

First, we assume that each carrier submits 4 requests to the auction pool. The reason is that for this number of requests it is still possible to run the auction with the complete set of bundles. With a pool size of 12 requests, the auctioneer can build up to 4095 bundles. In Table 3, we compare the results obtained with this complete bundle pool to those obtained with a limited number of bundles. For all experiments, where limited numbers of bundles are produced, we use our GA-based approach. We generate auction pools with 50, 100, 200, 300, and 500 bundles. The number of bundles that are offered to the carriers is indicated as $\#bundles$ in the following tables. All carriers are offered the same set of bundles. Please note that for calculating the reported collaboration improvements, steps 3 and 4 of the auction procedure described in Sect. 1 have to be applied. In step 3, the carriers give their bids based on the marginal profit of each bundle. This means that for each bid they have to solve a routing problem. In step 4, we find the optimal solution for the WDP by completely enumerating all possible assignments.

**Table 3:** Comparison between complete and limited bundle pool, where carriers submit 4 requests to the pool

Instance	# Bundles	Result (%)	Avg. runtime
O1_10	4095		12.19
	50	$-$ 7.3	0.52
	100	0.0	0.65
	200	0.0	0.95
	300	0.0	1.27
	500	0.0	1.84
O2_10	4095		10.54
	50	$-$ 19.9	2.15
	100	$-$ 9.9	0.43
	200	$-$ 7.2	0.65
	300	$-$ 7.2	1.05
	500	$-$ 7.2	1.47
O3_10	4095		14.58
	50	$-$ 36.0	0.4
	100	$-$ 26.5	0.6
	200	$-$ 13.0	1.01
	300	$-$ 10.3	1.53
	500	$-$ 7.6	2.22
O1_15	4095		60.07
	50	$-$ 12.1	1.01
	100	$-$ 11.1	1.68
	200	$-$ 5.8	2.91
	300	$-$ 5.8	4.27
	500	$-$ 5.1	6.71
O2_15	4095		83.26
	50	$-$ 23.0	1.25
	100	$-$ 7.4	2.12
	200	$-$ 5.0	3.69
	300	$-$ 3.3	5.81
	500	$-$ 3.3	9.31
O3_15	4095		104.7
	50	$-$ 35.6	1.49
	100	$-$ 24.5	2.55
	200	$-$ 18.5	4.41
	300	$-$ 14.5	7.6
	500	$-$ 7.9	11.44

Column *Result* shows the average percentage deviation in collaboration improvement. A negative value implies that the collaboration improvement found with the limited bundle pool was less than that found with the complete bundle pool. Average runtimes are in seconds

We see that for instance set O1_10, the complete and the limited pool starting with 100 bundles lead to the same results. However, by limiting the pool size, the computational time is decreased significantly from 12.2 to 1 second on average. Obviously, putting more bundles in the pool leads to better results. On average, limiting the pool size to 500 bundles leads to a decrease in solution quality of only 5.2%, while the average computational time is reduced from 47.6 to 5.5 seconds. This is displayed in Table 4.

We further observe that when offering only a low number of bundles (50) the deviation in collaboration improvement is very sensitive to the degree of overlap of the customer regions (see Table 3). It is shown in Berger and Bierwirth (2010) that in strongly overlapping areas (O3_10 and O3_15 in our instances), the collaboration profit is higher than in areas with less overlapping customer regions (O1_10 and O1_15 in our instances). Thus, if there is a high degree of overlap, many bundles are attractive. Being too restrictive with the number of offered bundles has a major impact on the solution quality, in the sense that it is hard to explore the huge solution space efficiently. Clearly, if the customer overlap is high, it is very easy to find good collaboration profits also with few bundles.

It should be mentioned that the performance of our method was compared to a setting, where the auctioneer offers randomly generated bundles to the carriers. We do not report these results in detail, because this random strategy could by far not reach the solution quality of our GA-based framework. For the smallest instances (O1_10) with 500 bundles, our method yields an average percentage deviation in collaboration improvement of 84.97%, while with random bundles we reach only 0.26%. In total, i.e., considering all instances and 500 bundles, the average percentage deviation in collaboration improvement is 57.72% (GA) vs. 2.43% (random).

**Table 4:** Comparison between complete and limited auction pool in average solution quality and average runtime

# Bundles	Avg. result (%)	Avg. runtime
4095		47.6
50	$-$ 22.3	1.1
100	$-$ 13.3	1.3
200	$-$ 8.2	2.3
300	$-$ 6.9	3.6
500	$-$ 5.2	5.5

In the next table, the number of requests each carrier is allowed to submit is set to 5. This increases the theoretical pool size to $2^{15}-1$, i.e., 32767 bundles. The results for the smaller instances O1_10, O2_10, and O3_10 are given in Table 5. This number of bundles cannot be evaluated within reasonable computational time, since it would imply that in the bidding phase each carrier has to solve 32767 routing problems. However, we see that the limited auction pools lead to very good results. With a pool size of 500, the average deviation from the result obtained with all bundles (4 requests per carrier) is 0.8%. For instances O1_10, the limited pools of 300 and 500 bundles even find better results than the complete pool (4 requests per carrier).

**Table 5:** Comparison between complete (4 requests per carrier) and limited (4 and 5 requests per carrier) pools for smaller instances (O1_10-O3_10)

Instance	# Bundles	# Req. per carrier (%)	
		4	5
O1_10	$2^n-1$		n/a
	100	0.0	$-$4.8
	300	0.0	0.2
	500	0.0	0.2
O2_10	$2^n-1$		n/a
	100	$-$9.9	$-$6.5
	300	$-$7.2	$-$4.9
	500	$-$7.2	$-$2.4
O3_10	$2^n-1$		n/a
	100	$-$26.5	$-$14.5
	300	$-$10.3	$-$3.1
	500	$-$7.6	$-$0.2
Average	100	$-$12.2	$-$8.6
	300	$-$5.9	$-$2.6
	500	$-$4.9	$-$0.8

*na* indicates that the complete pool size of these instances could not be solved. The numbers are the average deviation from the results obtained with the complete pool (4 requests per carrier). Positive values indicate that the limited pools found better results than the complete pool

The results for the larger instances (O1_15, O2_15, O3_15) are displayed in Table 6. Again we yield very good results with the limited auction pools. If each carrier submits 5 requests, even with a pool of only 300 bundles we obtain better results than with the complete pool (4 requests per carrier). With 500 bundles, the average improvement is 3.9%.

If carriers are allowed to submit up to 10 requests, and a pool of 500 bundles is composed, the results are 39.4% better than with the complete pool (4 requests per carrier).

It is obvious that a larger pool of requests (5 or 10 submitted requests instead of 4) comes with a different solution space, i.e., a potentially increased collaboration potential. However, this part of our computational study shows that solution quality increases significantly, even if the set of offered bundles is limited to a relatively small number. Hence, the results in Tables 5 and 6 show that it is worth to offer a limited set of attractive bundles, rather than to offer all bundles but being restricted to a relatively small number of traded requests.

**Table 6:** Comparison between complete (4 requests per carrier) and limited (4 and 5 and 10 requests per carrier) pools for larger instances (O1_15-O3_15)

Instance	# Bundles	# Req. per carrier (%)		
		4	5	10
O1_15	$2^n-1$		n/a	n/a
	100	$-$11.1	5.8	49.6
	300	$-$5.8	10.2	57.9
	500	$-$5.1	10.8	60.4
O2_15	$2^n-1$		n/a	n/a
	100	$-$7.4	$-$8.8	19.7
	300	$-$3.3	4.0	36.8
	500	$-$3.3	5.8	38.9
O3_15	$2^n-1$		n/a	n/a
	100	$-$24.5	$-$23.50	2.6
	300	$-$14.5	$-$9.24	20.4
	500	$-$7.9	$-$4.88	18.9
Average	100	$-$14.4	$-$8.8	24.0
	300	$-$7.9	1.7	38.4
	500	$-$5.4	3.9	39.4

*na* indicates that the complete pool size of these instances could not be solved. The numbers are the deviation from the results obtained with the complete pool (4 requests per carrier). Positive values indicate that the limited pools found better results than the complete pool

By the efficient reduction of the bundle pool, we are now able to handle instances of real-world size (e.g., Montoya-Torres et al. 2016; Lin 2008; Sprenger and Mönch 2012). Table 7 displays the runtimes of 3 randomly generated instances (O1_70, O2_70, and O3_70) with 210 requests. In the pool, there are 45 and 90 requests. These instances can be run on average in about 1 hour. We observe that a major part of this computational time goes to the bidding procedure.

**Table 7:** Runtimes in minutes of instances with 70 requests per carrier

Instance	# Bundles	# Req. per carrier	
		15	30
O1_70	$2^n-1$	n/a	n/a
	500	53.14	64.5
O2_70	$2^n-1$	n/a	n/a
	500	42.14	71.41
O3_70	$2^n-1$	n/a	n/a
	500	40.57	77.70
Average	500	45.29	71.20

## Conclusion

In this study, we investigated the bundling problem in combinatorial transportation auctions. Since the number of possible bundles increases exponentially in the number of traded requests, real-world settings can only be run if the auction pool is reduced to a subset of possibly attractive bundles. However, since carriers do not reveal sensitive information, the auctioneer has to compose these subsets having only incomplete information. In particular, the attractiveness of a bundle cannot be determined exactly, since marginal profits will not be disclosed.

We defined and modeled the BuGP, in which the aim is to provide the set of offered bundles that maximizes the total marginal profit. However, the objective function could only be evaluated whether carriers revealed sensitive information. Therefore, we presented a proxy for the objective function which is used to assess the attractiveness of bundles under incomplete information. In a GA-based framework, we apply this proxy to produce attractive and feasible bundles, such that all requirements of the BuGP are met. Although the carriers do not reveal sensitive information, we can guarantee that a feasible assignment of bundles to carriers is found.

We compared the results obtained by the limited set of bundles to those of the complete bundle pool. This showed that with only a small loss in solution quality, instances can be solved in a fraction of the computational time. Our approach enabled us to run and analyze instances that were previously intractable due their computational complexity. We found that, being able to increase the number of traded requests from 4 to 10, significantly higher collaboration profits can be achieved, even if the set of offered bundles is limited to 100.
